# Analysis of intra- and inter-observer variability in 4D liver ultrasound landmark labeling

**DOI:** 10.1117/1.JMI.12.5.051807

**Published:** 2025-06-30

**Authors:** Daniel Wulff, Floris Ernst

**Affiliations:** a Universität Rostock, Rostock, Germany; b Universität zu Lübeck, Lübeck, Germany

**Keywords:** tracking, liver, motion compensation, radiotherapy

## Abstract

**Purpose:**

Four-dimensional (4D) ultrasound imaging is widely used in clinics for diagnostics and therapy guidance. Accurate target tracking in 4D ultrasound is crucial for autonomous therapy guidance systems, such as radiotherapy, where precise tumor localization ensures effective treatment. Supervised deep learning approaches rely on reliable ground truth, making accurate labels essential. We investigate the reliability of expert-labeled ground truth data by evaluating intra- and inter-observer variability in landmark labeling for 4D ultrasound imaging in the liver.

**Approach:**

Eight 4D liver ultrasound sequences were labeled by eight expert observers, each labeling eight landmarks three times. Intra- and inter-observer variability was quantified, and observer survey and motion analysis were conducted to determine factors influencing labeling accuracy, such as ultrasound artifacts and motion amplitude.

**Results:**

The mean intra-observer variability ranged from 1.58  mm±0.90  mm to 2.05  mm±1.22  mm depending on the observer. The inter-observer variability for the two observer groups was 2.68  mm±1.69  mm and 3.06  mm±1.74  mm. The observer survey and motion analysis revealed that ultrasound artifacts significantly affected labeling accuracy due to limited landmark visibility, whereas motion amplitude had no measurable effect. Our measured mean landmark motion was 11.56  mm±5.86  mm.

**Conclusions:**

We highlight variability in expert-labeled ground truth data for 4D ultrasound imaging and identify ultrasound artifacts as a major source of labeling inaccuracies. These findings underscore the importance of addressing observer variability and artifact-related challenges to improve the reliability of ground truth data for evaluating target tracking algorithms in 4D ultrasound applications.

## 1 Introduction

Ultrasound (US) imaging is widely used in clinics for diagnosis as well as therapy guidance. It allows to visualize soft tissue structures in real time without the drawback of using harmful radiation. These characteristics and the capability of acquiring volumetric images make four-dimensional (4D) US imaging a promising modality for therapy guidance in radiotherapy.^1^^–^^3^ As tumors can undergo motion caused by physiological processes such as breathing or arbitrary patient movements, the exact tumor position must be tracked continuously to ensure that the tumor is treated as planned. Especially, abdominal tumors are affected by breathing-induced motion which leads to translation, rotation, and deformation.^2^^,^^4^ These high-dimensional motion patterns highly complicate tumor tracking in 4D US. Thus, research is in progress to develop robust and real-time capable target-tracking methods. Different approaches such as block-matching,^5^ feature-based tracking,^6^ shape models,^7^ or deep learning approaches^8^^,^^9^ have been proposed. Each approach was tested using expert-labeled US data. Expert labeling is a common method for creating ground truth data in US imaging. Expert-labeled US datasets exist in the literature for different anatomical domains provided with segmentations^10^^–^^12^ or landmarks.^13^ However, labeling variability is rarely determined. In Ref. 10, the inter- and intra-observer variability is determined for segmentations in two-dimensional US images. For three-dimensional (3D) US images, there is a lack of available datasets and labeling variability investigation. Only the Challenge on Liver Ultrasound Tracking (CLUST) dataset^13^^,^^14^ is available provided with inter-observer variability. Other approaches use implanted markers that can be tracked with an electromagnetic system such as Calypso (Varian Medical Systems, Palo Alto, California, United States) or creating synthetic data, e.g., using generative adversarial networks^15^ bring some drawbacks: implanted markers are clearly visible in US images, which can strongly affect the tracking algorithms executed on the US images.^16^ Using synthetic data, high-quality ground truth data can be generated. However, creating highly realistic synthetic US images is challenging. Furthermore, algorithm evaluation on real data is still required after testing on synthetic data to investigate the usability of the developed algorithms. Due to these challenges, expert labeling is the gold standard for creating ground truth data for US images. Even though expert labeling is common, this approach also brings some drawbacks. Finding experts to interpret US images can be challenging due to their rarity. Even when an expert is found, it is important to keep in mind that they are human and may make mistakes, resulting in minor inaccuracies. Therefore, landmark labels provided by an expert may be affected by inaccuracies. Furthermore, it might be possible that another expert observer would set labels slightly differently when labeling a landmark in a US image. Even though expert labeling is the gold standard, there is a lack of observer variability investigation for 4D US labeling. In this study, an intra- and inter-observer variability study is performed to quantify the labeling accuracy of experts in 4D US image data.

## 2 Methods

For determining the observer variability, a 4D US dataset and eight expert observers are available. In the following, the preparation of the US dataset as well as the labeling tool and the labeling procedure performed by the observers are described.

### 2.1 4D Ultrasound Dataset

The dataset used in this study contains long-term 4D US sequences (time-resolved 3D US images) of the liver from four different healthy subjects acquired by Ref. 17. All subjects were male aged 27 to 38 years. The US data were acquired under robot-guided motion compensation by attaching the US probe to a force-sensitive LBR iiwa 7 R800 (KUKA, Augsburg, Germany) robot. An EPIQ7 US system with an *X*6-1 matrix array US probe (Philips Healthcare, Best, The Netherlands) was used for data acquisition. The subjects lay on a treatment table while the robotic 4D US system acquired US images continuously for ∼30  min at a frame rate of ∼4  Hz. The US probe was placed in a lateral intercostal viewport on the subjects. For the labeling study, two sub-sequences are defined in each of the four long-term 4D US sequences with a temporal distance of ∼10  min. Thus, eight US sequences are prepared in total. Details about the sequences are summarized in Table 1.

**Table 1:** Characteristics of the available US sequences.

Sequence	Frame rate (Hz)	No. of frames	Length (s)	Voxel spacing (${\mathrm{mm}}^{3}$)	Volume size (vx)	Landmarks to label
V1-1	3.5	106	32	1.1×0.8×1.6	256×260×133	L1a and L1b
V1-2	3.5	108	31	1.1×0.8×1.6	256×260×133	L1a and L1b
V2-1	4.5	136	31	1.0×0.7×1.4	240×245×117	L2a and L2b
V2-2	4.5	136	29	1.0×0.7×1.4	240×245×117	L2a and L2b
V3-1	4.0	120	27	1.0×0.7×1.4	240×246×126	L3a and L3b
V3-2	4.0	120	28	1.0×0.7×1.4	240 × 246 × 126	L3a and L3b
V4-1	3.5	106	31	1.1×0.8×1.6	256×260×133	L4a and L4b
V4-2	3.5	112	34	1.1×0.8×1.6	256×260×133	L4a and L4b

The sequences have a length of 30 s, on average, and contain 3D US images with an average size of 248×253×127  vx.

### 2.2 Labeling Tool

**Fig. 1:**
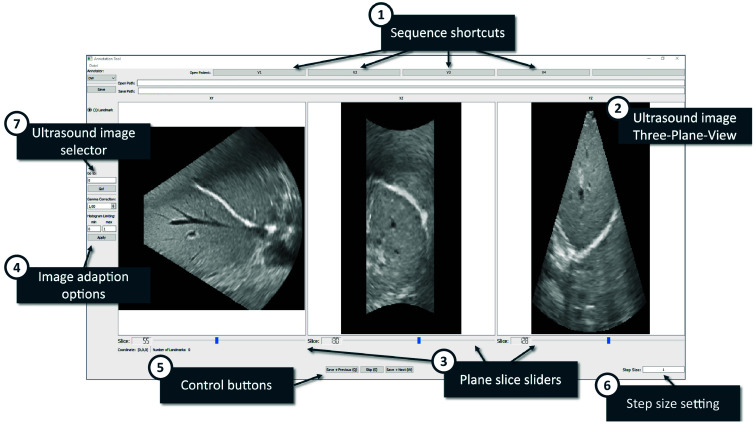
Screenshot of the labeling tool GUI used for 4D US labeling. The most important parts of the tool are image loading (1), US image visualization (2), control (3, 5, 6, 7), and image adaption (4).

To prepare a simple and easy-to-use labeling pipeline, a labeling tool was implemented. The tool has a graphical user interface (GUI) that aims to support the observers setting landmark labels within a 3D US image. Using a custom tool provides a high level of flexibility when setting up an efficient labeling pipeline, as well as being simple to use. A labeling pipeline was implemented, visualizing the subsequent US image after the previous image had been labeled. This automated pipeline eliminates the need for users to manually load US images and store landmarks, saving them time. The GUI and the functionality of the tool, visualized in Fig. 1, are implemented in Python using Qt5 and can be made available on request.

The tool consists of seven main parts that are marked in Fig. 1 and described in the following.

1. For loading one of the 4D US sequences described above easily, sequence shortcuts are placed in the upper part of the tool GUI. By pushing a sequence shortcut, the first image of the ultrasound sequence from the corresponding volunteer is loaded and visualized. As two sequences come from the same volunteer (e.g., V1-1 and V1-2) and the same landmark has to be labeled, these sequences are visualized directly one after the other.
2. The 3D US image is displayed in a three-plane view showing the xy-, xz-, and yz-planes of the image. This type of view was chosen as it is common and the experts had experience with it. With this view, the user is able to examine the image along all three volume axes, supporting the user in analyzing the image and locating the anatomical landmark to be labeled. Furthermore, the user performs landmark labeling in the displayed views by clicking directly at the position the user wants to label. The labeled landmark can be adjusted by clicking on a new location.
3. To be able to adapt the slices visualized in the three-plane view, for each plane, a slider is available located under the views. The current slice number is displayed next to the slider for easier orientation. In addition, the slices can be changed using the mouse wheel.
4. US image quality can be affected, e.g., by poor contrast or brightness. To compensate for such effects, two point-operation image adaption methods are added to the labeling tool: gamma correction and histogram clipping. These functionalities can be used to adapt the US image visualization to increase the visibility of the anatomical landmark to be labeled. The gamma correction defined by
  $$f_{\gamma }(I,\gamma )=I^{\gamma },$$ (1)
  maps every voxel value in the image I∈[0,1] to a new value in the range [0,1]. For this, the user can set the parameter γ∈R. By default, γ=1. Histogram clipping is defined by
  $$f_{\mathrm{clip}}(I,g_{\min},g_{\max})=\{\begin{array}{ll} 0 & \mathrm{if}\;0<I<g_{\min}\\ \frac{I-g_{\min}}{g_{\max}-g_{\min}} & \mathrm{if}\;g_{\min}\leq I\leq g_{\max}\\ 1 & \mathrm{if}\;g_{\max}<I\leq 1 \end{array},$$ (2)
  where $g_{\min},g_{\max}\in [0,1]$ and $g_{\min}<g_{\max}$ define the minimum and maximum grayscale values for clipping. By default, $g_{\min}=0$ and $g_{\max}=1$.
5. For labeling a whole US sequence, the images are visualized one after another by the tool. The user can use the control buttons located in the lower part of the tool GUI to save the annotated landmark and go to the previous or next US image of the sequence. This provides an easy-to-use labeling procedure for time-resolved US data without the time-consuming need of opening every US image manually.
6. Labeling every 3D US image of the sequence is time-consuming. To reduce the costs, a step size parameter τ∈N can be set in the lower right part of the tool GUI. This parameter defines the temporal step size that is applied when loading the next US image. By default, τ=1.
7. If the user wants to open a certain frame of the US sequence, this can be done by typing the frame number into the field on the left of the GUI and pushing the button “Go!”. The label that has been selected will be saved, and the desired US image will be loaded and visualized.

This labeling tool is used to label the eight liver US sequences defined above.

### 2.3 Sequence Labeling

Eight observers with expert knowledge in medical image analysis and processing with a mean working experience with 3D US image data of 1.8 years are available for labeling. The observers are randomly grouped into the groups A={A1,A2,A3,A4} and B={B1,B2,B3,B4}. In addition, in every sequence, two landmarks to be labeled are defined according to suggestions by the observers. As can be seen in Table 1, in each sequence, one landmark is assigned to observer group A and the other one to group B. Group A was asked to label the landmarks L1a, L2a, L3a, and L4a. Observer group B was asked to label the landmarks L1b, L2b, L3b, and L4b. Prior to the labeling process, the observers inspected a small subset of the dataset using the annotation tool to familiarize themselves with the dataset and the tool. In addition, the observers suggested different landmarks. In each US sequence, the two most suggested landmarks were selected as landmarks to be labeled. In the study, all observers were asked to label these pre-defined landmarks in the given US sequences three times each. However, as the labeling process is time-consuming, the effort is reduced by considering only every second US image. A labeling session consists of labeling the given landmark in every second US image of the sequence (parameter τ=2) using the tool described above. The observers had to finish labeling a sequence before starting with another one to reduce the risk of labeling inaccuracies due to breaks in the labeling process. The ultrasound sequences and the landmark labels created in this study are available on request. In addition, the observers were asked to answer a survey after performing labeling to evaluate how they rate the level of difficulty of the labeling process. The survey was filled once for each landmark by all observers and contained the questions presented in Table 2.

**Table 2:** Questions of the survey the observers had to answer for every landmark after labeling.

ID	Question	Answer range	
		From	To
Q1	Rate the level of clarity according to the landmark definition.	1: very clear	5: very unclear
Q2	Rate how much the target was clearly visible in the US sequence.	1: always visible	5: rarely visible
	Rate how much the level of labeling difficulty was affected by…		
Q3	…image noise.	1: not at all	5: very much
Q4	…US artifacts.	1: not at all	5: very much
Q5	…poor image contrast.	1: not at all	5: very much
Q6	…rotational target motion.	1: not at all	5: very much
Q7	What image adaption options did you use during the labeling procedure?	None, histogram clipping, and gamma correction	

Questions Q1 to Q6 had to be answered by rating on a five-point Likert scale. In question Q7, the observers had to name the image adaption methods they used during the labeling process (gamma correction, histogram clipping, or none).

## 3 Evaluation

The landmark labels provided by the experts are evaluated in terms of intra- and inter-observer variability. In addition, the survey is evaluated and interpreted together with the variability results.

### 3.1 Intra- and Inter-Observer Variability

For measuring observer variability, different ways and metrics have been described in the literature.^18^^–^^20^ In the domain of labeling 3D US image data, Ref. 14 used distance measurements among landmark labels for measuring observer variability. Thus, to achieve comparable results, the labeling error between two landmark labels $x,y\in R^{3}$ is determined using the Euclidean distance defined by

$$d_{\mathrm{error}}(x,y)=∥x-y{∥}_{2}.$$ (3)

Quantification of the labeling accuracy is performed by measuring the intra- and inter-observer variability. Here, the intra-observer variability is a measurement for the repeatability, and the inter-observer variability is a measurement for the reproducibility of labeling a landmark in 3D US images.^20^ To get one variability measurement from a set of several observations $L=\{l_{1},\ldots ,l_{n}\}$, a set of all possible pairwise distances is calculated by

$$D_{\mathrm{error}}=\{d_{\mathrm{error}}(l_{i},l_{j})\;|i,j\in \{1,\ldots ,n\},i<j\}.$$ (4)

Based on this set of distances, the mean observer variability and the standard deviation (SD) are determined.^21^ This procedure is done separately for both calculating the intra- and the inter-observer variability. For intra-observer variability, all observations from the same observer are used (three labels per observer), and for inter-observer variability, all observations from all observers who labeled the same landmark are used (four observers per landmark).

#### 3.1.1 Intra-observer variability

**Fig. 2:**
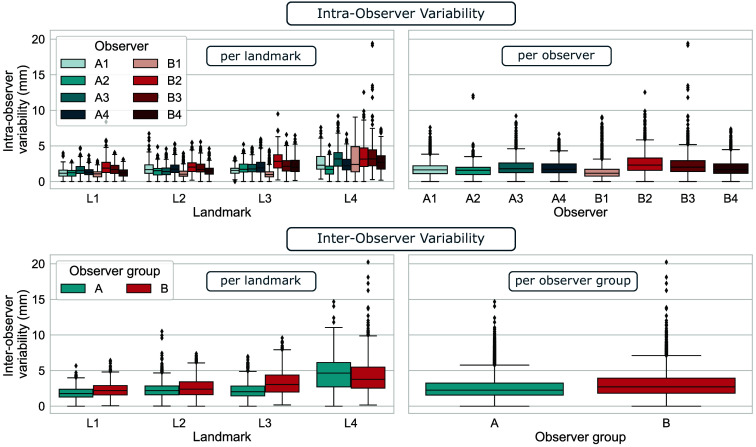
Intra- (top) and inter-observer (bottom) variability results depending on the landmarks (left) and on the observers (right) measured in this study.

The results of the study are summarized in Fig. 2. The intra-observer variability results are presented in the top row and the inter-observer variability results in the bottom row. As illustrated in Fig. 2, the mean intra-observer variability varies depending on the observer (min, max) = (1.58  mm±0.90  mm, 2.05  mm±1.22  mm) and the landmark (min, max) = (1.06  mm±0.54  mm, 3.53  mm±2.29  mm). In the labels from observer B3, e.g., variability outliers of up to 19.40 mm, were observed, whereas observer A4 had a maximum variability value of 6.67 mm. The highest intra-variability values are measured for the landmarks L4a and L4b with mean intra-observer variability of 2.53  mm±1.38  mm and 3.28  mm±2.04  mm, respectively. In contrast to that, for the landmarks L1a and L1b mean intra-observer variability of 1.34  mm±0.68  mm and 1.52  mm±0.88  mm are measured, respectively. Thus, a mean intra-observer variability difference of up to 1.94 mm is measured depending on the landmark and the sequence to be labeled.

#### 3.1.2 Inter-observer variability

**Fig. 3:**
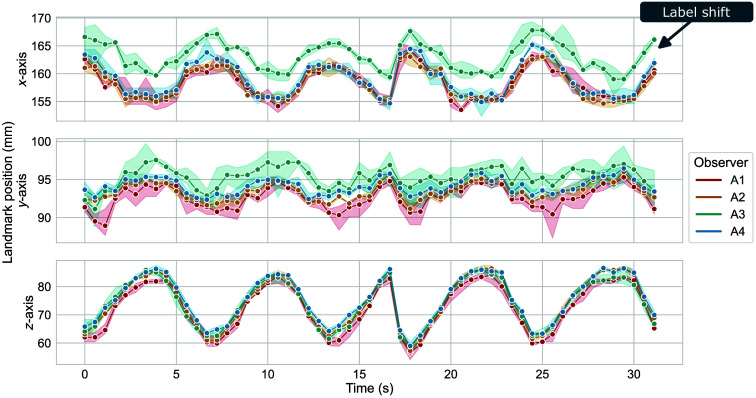
Landmark labels provided by the four observers of group A for the sequence V4-1 along the x-, *y*- and *z*-axes of the 3D US image. Observer A3 produced a label shift in the *x*- and *y*-axes in comparison with the remaining observers labeled with blue box leading to high inter-observer variability of 4.54  mm±2.16  mm.

Furthermore, it is observed that the mean inter-observer variability with 2.68  mm±1.69  mm in group A and 3.06  mm±1.74  mm in group B is higher than the intra-observer variability. This indicates that initially locating the landmark to be labeled was more challenging for the observers than repeating the landmark annotation. However, when considering the mean label position determined from the three label positions set by one observer, the mean inter-observer variability decreases significantly (p-value≪0.01, applying an independent *t*-test). The mean inter-observer variabilities of 2.20  mm±1.49  mm and 2.51  mm±1.31  mm are measured in groups A and B, respectively. The 95% confidence interval (95% CI) for the groups A and B are [0.42,0.56] and [0.49,0.62], respectively. The inter-observer variability also depends on the landmark itself, as can be seen in Fig. 2 (bottom left). For instance, the mean inter-observer variability for landmark L1a is 1.85  mm±0.78  mm, whereas it is 4.54  mm±2.16  mm for L4a. It could be observed that observer A3 labeled the landmark L4a 5.25 mm apart from the labels of the other observers, on average. This is caused by a labeling shift as presented in Fig. 3.

Here, it can be seen that the labels provided by observer A3 are shifted mainly along the *x*- and *y*-axes of the image, in comparison with the labels set by the remaining observers of group A. In total, such a label shift could be observed for three out of eight landmarks, namely, L2b, L3b, and L4a. However, all these label shifts were produced either by observers A3 or B2. This indicates that the landmark definition was not always clear enough for these observers.

### 3.2 Observer Survey

**Fig. 4:**
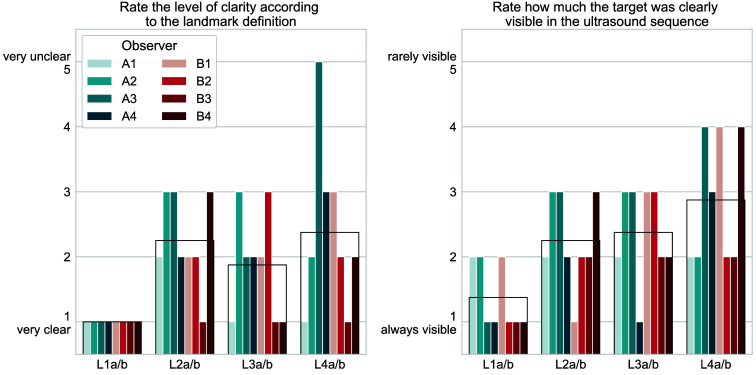
Observer ratings for the questions Q1: “Rate the level of clarity according to the landmark definition” and Q2: “Rate how much the target was clearly visible in the US sequence” on a five-point Likert scale for all US sequences. The average values are marked black.

After labeling a landmark in a whole 4D US sequence, the observers were asked to fill in a survey containing the questions presented in Table 2. Every observer filled out the survey once for the landmarks L1, L2, L3, and L4 each, meaning four times in total. The questions Q1 and Q2 focus on the target definition and visibility in the US sequences. The observers’ answers are presented in Fig. 4.

**Fig. 5:**
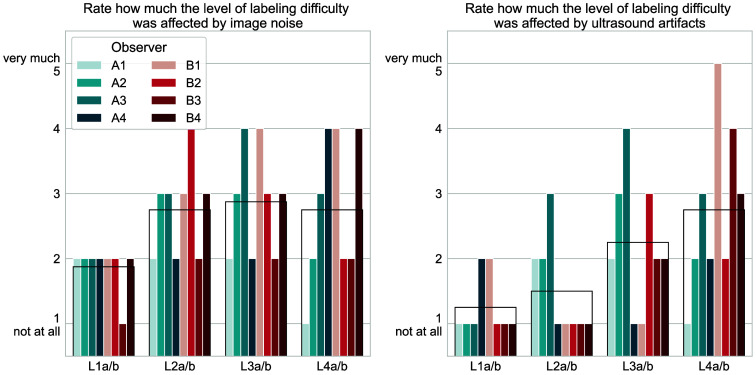
Observer ratings for the questions Q3: “Rate how much the level of labeling difficulty was affected by image noise” and Q4: “Rate how much the labeling difficulty was affected by US artifacts” on a five-point Likert scale for all US sequences. The average values are marked black.

**Fig. 6:**
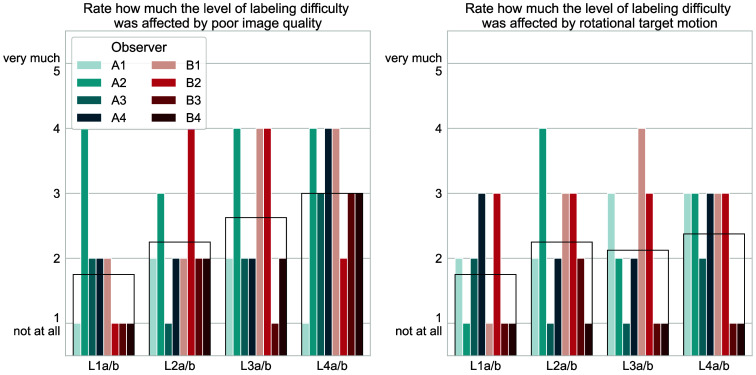
Observer ratings for the questions Q5: “Rate how much the level of labeling difficulty was affected by poor image contrast” and Q6: “Rate how much the labeling difficulty was affected by rotational target motion” on a five-point Likert scale for all US sequences. The average values are marked black.

It can be seen that only once (observer A3 for landmark L4a) the landmark definition was rated 5 (very unclear). This low level of landmark definition clarity could be a reason for the label shifts shown before. However, in total, the landmark definition was rated 1 (very clear) or 2 in 75% of the answers. This indicates that most of the landmarks to be labeled were intelligibly defined for the observers. The visibility of the target within the US sequences (question Q2) was rated 1 (always visible) or 2 in 66% of the cases. No observer rated a landmark to be rarely visible. Thus, the targets were predominantly visible to the observers. However, it can be seen that the level of target visibility for the landmarks L4a and L4b is rated lower than for the other landmarks, indicating that these landmarks were harder to label for the observers. This finding could be a reason for the higher intra-observer variability values measured for the landmarks L4a and L4b. In questions Q3 to Q6, the observers had to rate how much image noise, US artifacts, image contrast, and rotational target motion affected the level of labeling difficulty. The observers’ answers are presented in Figs. 5 and 6.

It can be seen that image noise affects the labeling difficulty for all observers in all US sequences. Only two times the observers answered question Q3 with 1 (not at all). In contrast, US artifacts highly affected the labeling difficulty for the landmarks L3a, L3b, L4a, and L4b as the observers answered this question with an average rating of 2.4 for these landmarks. For the remaining landmarks, image artifacts only had a minor impact on the labeling difficulty with an average rating of 1.4. The observers reported a rib shadow temporarily overlaying the landmarks L4a and L4b. This artifact occurred due to a rib moving into the field of view of the US probe caused by breathing-induced motion. As the landmark was overlaid by a shadow artifact, labeling the landmark became very challenging due to the limited visibility of the landmark. This finding could be the reason for the high rarely visible ratings for landmarks L4a and L4b and thus for the higher intra-variability values measured for these landmarks. The influence of the image quality (question Q5, Fig. 6) was rated differently by the observers depending on the landmarks. A similar trend and mean ratings for the influence of ultrasound artifacts (question Q4) can be observed. However, for the influence of rotational target motion (question Q6), no trend was measured. The ratings range from 1 (not at all) to 4 and are 2.1 on average.

To determine the factors that most influenced the observers’ landmark labels, the correlation between the intra-observer variability and the survey answers is calculated for every observer. For this, the Pearson correlation used is defined as

$$r(x,y)=\frac{\overset{n}{\underset{i=1}{\sum }}(x_{i}-\overset{¯}{x})(y_{i}-\overset{¯}{y})}{\sqrt{\overset{n}{\underset{i=1}{\sum }}(x_{i}-\overset{¯}{x}{)}^{2}}\sqrt{\overset{n}{\underset{i=1}{\sum }}(y_{i}-\overset{¯}{y}{)}^{2}}},$$ (5)

where $\bar{x}$ and $\bar{y}$ are the mean values of the data vectors x and y, respectively. In total, four statistically significant correlations (two-sided p-value<0.05) are determined. For observer A4, a correlation coefficient of 0.95 is measured between the landmark definition clarity (question Q1) and the intra-observer variability (95% CI of [0.33,1.00]). Thus, the landmark definition clarity plays an important role in a labeling task. The intra-observer variability of the observers B1, B3, and B4 highly correlate with the influence of US artifacts (question Q4) with correlation coefficients of 0.96, 1.00, and 0.98, respectively (95% CI of [0.05,1.00], [0.77,1.00], and [0.37,1.00], respectively). This indicates that US artifacts have a major influence on landmark labeling accuracy.

In the last question, the observers had to name what image adaptation method they used during labeling the US sequences. It turned out that the observers used histogram clipping the most in 56% of the sequences. In 38% of the sequences, they did not use an image adaptation method, and only in 6% they used gamma correction. In total, five out of eight observers used histogram clipping to adapt the US image visualization indicating that this method can be helpful when labeling US images.

### 3.3 Landmark Motion

To quantify the motion extent of the 4D US dataset, the landmark labels set by observer B1 were evaluated. This observer is chosen due to the fact that the lowest intra-observer variability is measured. As can be seen in Fig. 3, the landmarks move in a periodic sinusoidal way due to the respiration process. Thus, for determining the motion extent, the peaks of the inhale and exhale motion phases are detected in the landmark labels. The motion extent is defined as the difference between the landmark positions of the inhale and exhale phases. The mean Euclidean distance between these landmark positions is 11.56  mm±5.86  mm with a range of 4.92 mm to 31.25 mm. It could be observed that the motion extent of landmark L1b is much larger than the extent of the other landmarks. For L1b, a mean motion extent of 29.28  mm±0.91  mm is measured, whereas L4b has a mean motion extent of 12.77  mm±2.49  mm. For the remaining landmarks, even lower motion is determined. A correlation analysis between the landmark motion and the intra-observer variability is performed to examine whether high landmark motion affects labeling accuracy when landmarks have different motion extents. However, no correlation could be determined indicating that the landmark motion extent has no influence on labeling accuracy.

## 4 Discussion

The goal of this study was to measure the intra- and inter-observer variability in landmark labeling in 4D US. This information can assist in evaluating the performance of target-tracking algorithms by taking labeling inaccuracies into account. A labeling study is conducted and evaluated by measuring the variability of eight annotators, split into two groups. To the best of our knowledge, this is the first landmark labeling variability study conducted for 4D US. Intra-observer variability is a measure of repeatability, meaning how robust an observer can annotate a target. Thus, for evaluating the reliability of labeled ground truth in a target tracking task, the intra-observer variability is more convincing than inter-observer variability. In the CLUST challenge, however, the inter-observer variability is used as a variability measure, and it is even calculated in a different way than in this study.^13^^,^^14^ The authors determined an inter-observer variability of 1.36  mm±1.14  mm, 1.27  mm±1.07  mm, and 1.19  mm±0.83  mm for three observers. The reason for having three inter-observer values is due to the way of calculating it. Instead of determining the distances between the labels of the observers, the authors calculated the distance to the mean label position determined from the labels of the three observers.^13^ Thus, for each observer, an individual variability measure is calculated. When applying this calculation to the labels acquired in this work, a mean inter-observer variability range of (min, max) = (1.33  mm±0.79  mm, and 2.05  mm±1.04  mm) is determined for eight observers, which is in the same order of magnitude as in the CLUST dataset. The highest inter-observer variability values are determined for observers A3 (1.93  mm±1.49  mm) and B2 (2.05  mm±1.04  mm), which are caused by label shifts, probably due to the lack of clarity in landmark definition. The observers were provided with an annotated three-plane view image of the landmark. Based only on this information, the observers had to label the landmark. Although the landmarks were selected based on the observers’ suggestions, some landmarks were not clearly defined enough for all observers. To avoid this systematic error in future studies, two different approaches could be used: (1) A landmark is labeled by only one observer for the whole dataset. This would eliminate the risk of inter-observer variability, but the total labeling workload for a landmark is one observer. However, the workload could be spread across all available observers by distributing the landmarks. Labeling could be done repeatedly to be able to determine the intra-observer variability. (2) All observers jointly define rules for the labeling process to ensure that labels are set according to the same rules. Using this approach, the labeling workload could be spread across all available observers by sharing subsets of the US sequences. The observers can complement each other, avoiding dependence on a particular observer for a particular landmark.

Another frequently investigated anatomical domain is fetal US. In Ref. 22, labeling variability for fetal facial landmarks in the 3D US was investigated based on three experts. They determined intra- and inter-observer variability of 1.01 and 1.60 mm, respectively. However, facial landmarks such as the nose, eyes, and ears are well-defined structures and may be easier to label than abstract structures such as the vessels. Other US annotation studies focus on segmentation labeling in fetal ultrasound images^23^^–^^25^ where variability was measured using Dice scores ranging above 0.80. For other anatomical domains, there is a lack of observer variability investigation in the literature.

The US image data labeled in this study were obtained from healthy volunteers. This study did not investigate whether observer variability is different when labeling patient data. The results suggest that expert observers should be able to label with observer-dependent variability when the landmark is clearly defined and continuously visible. Depending on the disease and its effects on the patient’s body, the landmark motion patterns may be different, for example, because the respiratory system is affected. Whether such effects affect the labeling accuracy needs to be investigated in a further study.

In the intra-observer evaluation of this study, low mean variability is measured. During the labeling process, observers were free to choose the order in which they labeled the sequences. However, as the order was not tracked, it is not possible to examine any potential practice effects. This is a limitation of the study. However, it turned out that especially the landmark visibility affects the labeling accuracy as the variability increased in sequences where the landmark is overlaid by a rib shadow artifact. From this finding, recommendations for US image acquisition and labeling can be derived. During image acquisition, it should be made sure that the risk of shadow artifacts is minimized, for example, by choosing optimal ultrasound viewports. Before starting the labeling process, the data could be pre-inspected to identify potential artifact-affected landmarks and to reject them. However, this process would be time-consuming. Other approaches could be applied during labeling: The experts could be asked to indicate the reliability of the landmarks by adding confidence values, or they could be asked to refrain from labeling landmarks that they cannot reliably identify. With this additional information, the landmark quality could be increased.

The mean motion amplitude of the labeled landmarks (11.56  mm±5.86  mm) is in a similar extent as the liver motion measured in other studies. For instance, Ref. 26 measured mean ± SD displacement of 3.00  mm±2.00  mm and 5.10  mm±3.10  mm in LR and AP directions and a mean 3D motion magnitude of 10.10 mm with a range of 2.40 to 19.40 mm. Investigating the correlation between the motion amplitude and the intra-observer variability showed that there is no correlation. This indicates that the amount of motion does not affect the observers’ labeling accuracy. Considering the goal of using the landmark labels as ground truth for evaluating target tracking algorithms in 4D US, the evaluation accuracy would be limited to the intra-observer variability. Tracking errors smaller than this would not be reliable because it would not be clear whether the error was due to labeling or tracking inaccuracy. To reduce this limitation, labels that have been repeatedly set by an observer could be merged. As the intra-observer variability is a measurement for repeatability, the labeling inaccuracy could be overcome, e.g., by determining the mean label position. This is confirmed by the significant (p-value≪0.01) reduction in inter-observer variability when the mean label positions are considered instead of the raw label positions. However, this procedure would increase the labeling effort for the experts. As mentioned before, observers should jointly define rules for the labeling process to avoid systematic errors. This provides the possibility to spread the labeling workload across all available observers by splitting the dataset into subsets resulting in a reduction of labeling effort. When choosing overlapping subsets, multiple labels will be available for a landmark that could be merged.

The custom annotation tool used in this study was minimalist and simple to use. It was easy for the observers to familiarize themselves with it, and no observer reported issues while using it. However, this study did not investigate whether the tool or the three-plane view visualization is optimal. This is a limitation of this study. Even though the tool used offers benefits such as flexibility and simplicity, additional functionality and view options might be able to further simplify the labeling process or improve labeling accuracy. For example, providing a 3D view with the option of manual rotation could help with high-dimensional motion. However, the highly variable nature of such a visualization could complicate the labeling process and increase the time required to label. As the focus of this study was to investigate inter- and intra-observer variability under the same circumstances, the impact of using different annotation tools could not be investigated. To determine the optimal tool and visualization, another study is required. In Ref. 27, such a study was conducted for the medical image domain showing that simplicity and familiarity are important factors when selecting the tool. The study also claimed that (semi-)automatic assistance tools could be beneficial to save time and increase labeling accuracy.^27^ The results of the observer survey showed that observers needed to adapt the image visualization using an adaptation method in 56% of the US images. This confirms the need for assistance methods in the annotation tool for optimal visualization of US image data. By implementing automatic AI-based methods, the handling could be simplified as the need for manual image adaptation would be eliminated. In addition, AI-based pre-labeling could speed up the labeling process, as the observer would only have to adjust the label, not set it from scratch.

## 5 Conclusion

Target tracking in 4D US is a challenging task under research that could be used for therapy guidance as in radiotherapy. Different approaches were proposed and evaluated on labeled 4D US data. However, the reliability of the tracking performance depends on the labeling accuracy of the ground truth. To measure intra- and inter-observer variability in 4D US, a labeling study is conducted in this paper. Mean intra-observer variability of 1.58  mm±0.90  mm to 2.05  mm±1.22  mm and mean inter-observer variability of 2.68  mm±1.69  mm (group A) and 3.06  mm±1.74  mm (group B) depending on the observers are measured. It is determined that the labeling process can be highly influenced by US artifacts affecting the intra-observer variability. Inter-observer variability is mainly influenced by a lack of clarity of the landmark definition leading to label shifts. However, this does not affect the intra-observer variability. It could also be shown that the motion amplitude does not have an effect on the labeling accuracy. This study shows that the labeling accuracy in 4D US is limited to observer-specific repeatability. Thus, to provide landmark labels as accurate as possible, it might be useful to let an observer label the same data several times and merge the labels, e.g., by determining the mean label position. As long as the observer tries to label the same landmark in each labeling session, this could overcome the repeatability error. A limitation of this study is the low amount and variability of US sequences. In a future study, this will be overcome by adding more US sequences from different subjects or patients.

## Data Availability

The ultrasound sequences and the labels generated in this study, as well as the labeling tool, are available on request.
